# Advanced genetic therapies for the treatment of Rett syndrome: state of the art and future perspectives

**DOI:** 10.3389/fnins.2023.1172805

**Published:** 2023-05-25

**Authors:** Michela Palmieri, Diego Pozzer, Nicoletta Landsberger

**Affiliations:** ^1^Rett Research Unit, Division of Neuroscience, San Raffaele Hospital (IRCCS), Milan, Italy; ^2^Department of Medical Biotechnology and Translational Medicine, Faculty of Medicine and Surgery, University of Milan, Milan, Italy

**Keywords:** Rett syndrome, gene therapy, genome-RNA editing, read-through therapy, nanoparticle

## Abstract

Loss and gain of functions mutations in the X-linked *MECP2* (methyl-CpG-binding protein 2) gene are responsible for a set of generally severe neurological disorders that can affect both genders. In particular, *Mecp2* deficiency is mainly associated with Rett syndrome (RTT) in girls, while duplication of the *MECP2* gene leads, mainly in boys, to the *MECP2* duplication syndrome (MDS). No cure is currently available for *MECP2* related disorders. However, several studies have reported that by re-expressing the wild-type gene is possible to restore defective phenotypes of *Mecp2* null animals. This proof of principle endorsed many laboratories to search for novel therapeutic strategies to cure RTT. Besides pharmacological approaches aimed at modulating MeCP2-downstream pathways, genetic targeting of *MECP2* or its transcript have been largely proposed. Remarkably, two studies focused on augmentative gene therapy were recently approved for clinical trials. Both use molecular strategies to well-control gene dosage. Notably, the recent development of genome editing technologies has opened an alternative way to specifically target *MECP2* without altering its physiological levels. Other attractive approaches exclusively applicable for nonsense mutations are the translational read-through (TR) and t-RNA suppressor therapy. Reactivation of the *MECP2* locus on the silent X chromosome represents another valid choice for the disease. In this article, we intend to review the most recent genetic interventions for the treatment of RTT, describing the current state of the art, and the related advantages and concerns. We will also discuss the possible application of other advanced therapies, based on molecular delivery through nanoparticles, already proposed for other neurological disorders but still not tested in RTT.

## Introduction

Rett syndrome (RTT; OMIM 312750) is an X-linked neurodevelopmental disorder that almost exclusively affects girls (Amir et al., 1999). With an incidence of 1 in 10,000 it represents the most common genetic cause of severe intellectual disabilities in females worldwide (Neul et al., 2010). Apparently normal at birth, typical (or classic) RTT patients seem to grow up fine through the first 6–18 months of life, then their neurological development arrests and a regression phase occurs leading to manifestation of distinct symptoms, including loss of speech, impaired motor skills, stereotypical hand movements, gait abnormalities and seizures, that together constitute the hallmarks of the disease. Autistic features and other severe clinical traits such as apnea, hyperventilation, scoliosis, weight loss and cardiac abnormalities are often seen in affected girls (Chahrour and Zoghbi, 2007). In addition, atypical RTT patients manifesting either milder or more severe clinical features also exist. Notably, in contrast to what was initially reported, *MECP2* mutant males also have been described and they generally display greater clinical harshness compared to females; they can experience mild mental retardation or die from severe neonatal encephalopathy (Neul et al., 2018).

Originally characterized in 1966 by Andreas Rett, an Austrian pediatrician who observed two girls having same unusual behavior, it was only in 1999 that the laboratory of Huda Zoghbi discovered that variations in the X-linked methyl-CpG-binding protein-2 (*MECP2*) gene are causative of RTT. Indeed, 90–95% of individuals with typical RTT and 70% of atypical cases are mutated in *MECP2*. Additionally, genetic changes in the X-linked Cyclin-Dependent Kinase-Like 5 (*CDKL5*; OMIM #300203) (Evans et al., 2005) or the Forkhead box G1 (*FOXG1*; OMIM #164874) (Philippe et al., 2010) genes have been related with atypical and rarely classic forms of RTT (Neul et al., 2010). Most of the mutations in *MECP2* are hypomorphic, thus leading to partial or complete “loss of function” of the protein; however hypermorphic mutations, associated with duplication of portions of Xq28 spanning the *MECP2* locus, have been related to a neurodevelopmental disorder called MeCP2 duplication syndrome (MDS) (van Esch et al., 2005). MDS predominantly affects males, who manifest severe intellectual disability, delayed psychomotor development, seizures, respiratory infection, feeding difficulties and progressive spasticity. They often die before 25 years of age because of frequent infections and neurological decline. Remarkably, while RTT is a sporadic disease, *MECP2* duplications are inherited with >90% penetrance from mothers, who carry the mutated copy of the gene on the silenced X chromosome (Ramocki et al., 2010). Eventually, *MECP2* mutations have been associated with neurodevelopmental disorders such as Angelman-like syndrome and Attention-Deficit Hyperactivity Disorders (ADHD), and occasionally with autism (Ramocki et al., 2009). Collectively, these results of molecular genetics prove that *MECP2* can cause a broad spectrum of neuropsychiatric disorders and intellectual disabilities that can be gathered as MeCP2-related disorders.

To date there is no cure for RTT and ongoing treatments are meant to alleviate disease symptoms. For example, medications are provided to mitigate breath irregularities and sleep problems while antiepileptic drugs are administered to relief patients affected by seizures (about 60%) (Vignoli et al., 2017). Other treatment options that overall improve the quality of life of RTT girls include occupational and physical therapy, scoliosis equipment and nutritional programs. About 70% of individuals with typical RTT may survive longer than 45 years old with appropriate medical and care management (Tarquinio et al., 2015).

Importantly, a breakthrough study by Adrian Bird’s teams proved that restoration of endogenous *Mecp2* expression in symptomatic hemizygous male and heterozygous female adult mice reversed many of the Rett-like phenotypes even at the late stages of syndrome progression (Guy et al., 2007). The reversal of disease condition has led the scientific community to consider gene replacement therapy as the most amenable strategy to cure RTT and MeCP2-related disorders (Gadalla et al., 2013, 2017; Garg et al., 2013; Sinnett et al., 2017). However, pre-clinical studies of gene therapy in RTT demonstrated that overexpression of MeCP2 resulted in severe neurological defects and liver damage in injected animals (Collins et al., 2004; Gadalla et al., 2013; Matagne et al., 2021; Li et al., 2023). For these reasons, in the last decades, novel strategies were explored to identify the most appropriate delivery vector that could finely transduce *MECP2* mainly in brain cells without triggering deleterious side-effects.

By describing gene therapy strategies and engineered vectors able to control MeCP2 expression, we will review and comment on the most recent progresses of genetic interventions; further, we will discuss the latest development of DNA/RNA editing approaches and reactivation of the *Mecp2* allele placed on the silenced X-chromosome and open to future approaches for molecular delivery through nanoparticles that are still not tested in RTT.

## Genetics of RTT: the *MECP2* gene and its pathogenic mutations

**Figure 1:**
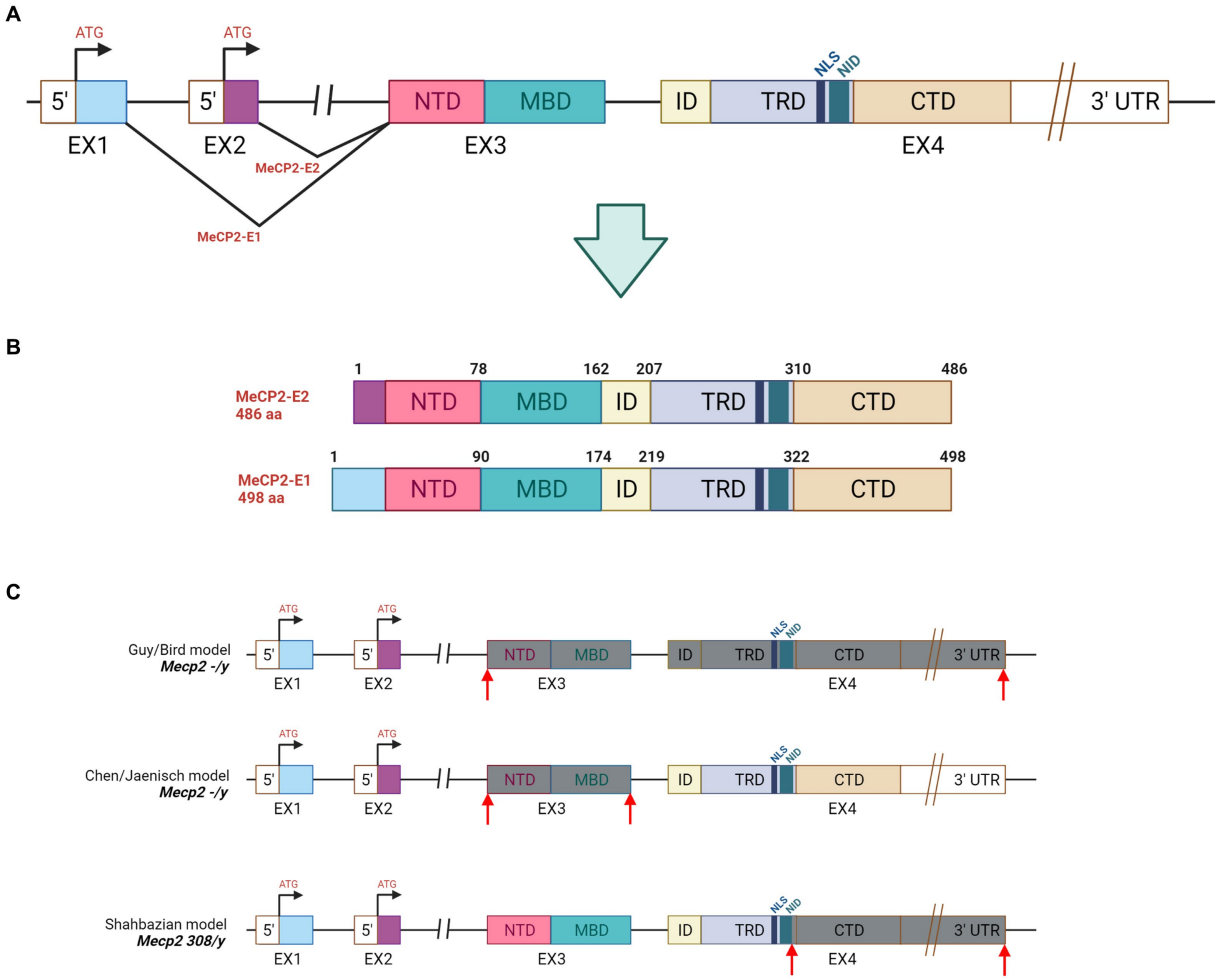
Gene and protein structure of MeCP2. **(A)** Gene structure: NTD = N-Terminal domain, MBD = Methyl-binding domain, ID = Intervening domain, TRD = Transciption repression domain, CTD = C-terminal domain, NID = NCoR intreraction, NLS = Nuclear localizatoin signal. **(B)** Illustration of MeCP2-E1 and MeCP2-E2 isoforms derived by alternative splicing in *MECP2*. **(C)** Genetic features of the mostly used mouse models of RTT. *Mecp2*^−/y^ mice were obtained by deleting the exon 3 and 4 (Guy et al., 2001) or by removing the exon 3 (Chen et al., 2001) from the *Mecp2* gene. The *Mecp2*^308/y^ animal model was produced by inserting the T308X nonsense mutation in exon 4. The resulting truncated protein lacks the C-terminal domain while maintaining the MBD and the TRD portions (Shahbazian et al., 2002). Grey boxes depict the exons that are missing in the MeCP2 protein, while the red arrows indicate the deleted portion.

The *MECP2* gene is located on the long arm of X-chromosome (Xq28) where it spans almost 76 kilobases (kb). Its 3′-UTR (untranslated region) is one of the longest known in the human genome as well as its second intron is atypically long (60,000 nucleotide) (Reichwald et al., 2000). *MECP2* is present in all vertebrates, and in both human and mouse consists of four exons from which two different protein isoforms are generated: the MeCP2 E1 and MeCP2 E2 (Figure 1A). In human, the MeCP2 E1 is encoded by exons 1, 3 and 4 while exon 2 is excluded via alternative splicing. The resulting product is a longer protein of 498 amino acid containing 21 unique N-terminal residues. The MeCP2 E2 is translated from exons 2, 3, and 4 and has 9 unique residues (Mnatzakanian et al., 2004) (Figure 1B). While MeCP2 E1 is conserved across vertebrates and predominantly expressed in adult brain, MeCP2 E1 is only present in mammals and highly expressed in peripheral tissues (Tillotson and Bird, 2019). Remarkably, recent evidence suggests that mutations in MeCP2 E1 might be associated with RTT (Yasui et al., 2014). The human MeCP2 E2 protein is structurally composed by 486 amino acid residues and functionally characterized by five main domains: N-terminal domain (NTD, 1–78 amino acid); methyl-CpG binding domain (MBD; 78–162) spanning 85-amino acid; intervening domain (ID); transcriptional repression domain (TRD) of 104- amino acid and carboxyterminal domain (CTD; 310–486 amino acid). MeCP2 binds to methylated cytosine via the MBD, while the CTD facilitates its interaction to naked or nucleosomal DNA thus mediating chromatin compaction (Figure 1B). Two other regions are mostly relevant, and both located in the TRD domain: the NCoR1/2 co-repressor complex interaction domain (NID, amino acid 285–313), a short region of 29-amino acid, and a nuclear localization signal (NLS; 255–271) of 16 residues. The estimated molecular weight of MeCP2 is approximately 53 kDa; however, in western blot the protein is detected at 72 kDa.

Over 500 different *MECP2* mutations have been identified as causative of RTT and documented in the web database (RettBase: http://mecp2.chw.edu.au). Among those, there are eight major point mutations (p.Arg106Trp (R106W), p.Arg133Cys (R133C), pThr158Met (T158M), p.Arg168* (R168X), p.Arg255* (R255X), p.Arg270* (R270X), p.Arg294* (R294X) and p.Arg306Cys (R306C)) that account for almost 65% of all variations found in typical RTT individuals (Neul et al., 2008). In addition, small deletions, predominantly falling in the C-terminal domain of MeCP2 are featured in 5–10% of classical RTT patients. Several studies supported the relationship between the clinical severity of RTT and the type of mutations in *MECP2* (Frullanti et al., 2019). Indeed, partial loss-of-function variations such as the R133C and late truncating mutations often lead to a milder phenotype, while missense mutations (e.g., R306C or T158M) and early truncating variations such as R294X are associated to phenotypes of medium severity. Complete loss-of-function mutations (e.g., R255X or other early truncations) and large deletions often yield to a very severe clinical course of RTT. Interestingly, mutations occurring within the NID region (e.g., R294X) destroy the interaction with transcriptional co-repressor complex NCoR/SMRT and disrupt the repressive activity of MeCP2. Similarly, variations falling in the MBD (e.g., R133C) partially abolish the binding to methylated DNA, thus confirming the biological relevance of these two domains (Cuddapah et al., 2014; Frullanti et al., 2019). However, other factors such as X-chromosome inactivation (XCI) participate to the phenotypic variability of RTT individuals. XCI defines the pattern of silencing of one X-chromosome that occurs in all cells during early female embryogenesis. Consequently, a female patient generally features half cells expressing the wild type *MECP2* allele and the other half the mutated one (random XCI). However, if this process is skewed and it favors the inactivation of mutant *MECP2* in most cells, no (as seen in silent carriers) or very mild clinical manifestations are overt; alternatively, the preferential inactivation of the wild-type allele causes an aggravation of the phenotype. Therefore, the degree of XCI skewness determines the greater clinical variability seen in RTT patients (Takahashi et al., 2008). Finally, genetic modifiers of *MECP2*, which mainly remain undisclosed, may also affect the variability of the disease.

In conclusion, the variability and clinical severity of RTT patients is the result of the complex combination among the type of *MECP2* mutation, the presence of genetic modifier(s), X-chromosome inactivation status, and environment.

## Neuron and glia: partners in RTT?

MeCP2 is ubiquitously expressed but its highest levels are reached in brain, lung and spleen. Its amounts are modest in heart and kidney and are almost undetectable in stomach and liver. In particular, in brain MeCP2 protein levels correlate with neuronal maturation, rising when neurons project dendritic arbors and axons, and when connectivity is established (Kishi and Macklis, 2004; Neul and Zoghbi, 2004). In addition, its expression remains high throughout adulthood thus supporting its main role of sustaining the activity of mature neurons. However, increasing evidence underlined its fundamental role in early stages of neuronal development (Ronnett et al., 2003; Sun et al., 2019). Indeed, MeCP2 expression was detected in embryonic and postnatal neocortical cells, including neuronal precursors (Kishi and Macklis, 2004; Bedogni et al., 2016). Further, transcriptional analyses of *Mecp2* null embryonic cortices revealed an enrichment of genes expressed by progenitors and early postmitotic neurons, and a decrease of transcripts involved in neuronal differentiation and responsiveness to external stimuli, thus providing another relevant role of MeCP2 in neuronal fate refinement and activity (Cobolli Gigli et al., 2018). However, the most conspicuous consequence of MeCP2 deficiency in the CNS is the reduced size and weight of the RTT brain (reduction of 12–34%). Other more subtle alterations include decreased dendritic complexity, defects in spine density and morphology, and increased neuronal packing (Armstrong et al., 1995; Bauman et al., 1995). *In vivo* studies performed on different mouse models showed that *Mecp2* deficiency also disrupts, with a pattern that varies among different brain regions, the balance of synaptic excitation and inhibition (Dani et al., 2005; Shepherd and Katz, 2011). Finally, long-term synaptic plasticity (i.e., Long Term Potentiation (LTP) and Long Term Depression (LTD)) which underlies the processes for cognitive functions and long-term memory formation is also generally affected (Li et al., 2016).

Even though MeCP2 was detected in many non-neuronal cell types, the neuropathology observed in RTT was exclusively ascribed to its loss in neurons. However, recent studies started to investigate whether glial cells might contribute to the RTT pathogenesis. Indeed, increased levels of genes encoding glia-specific proteins (e.g., α Β-crystallin, glial fibrillary acidic protein (GFAP), excitatory amino acid transporter 1 (EAAT1) and S100 A13) were documented in post-mortem brain of RTT girls (Colantuoni et al., 2001).

Consistently, transcriptional and proteomic analyses of *Mecp2* null brain cortices revealed a perturbation of pathways involved in astrocytic maturation and morphology (Yasui et al., 2013; Delépine, 2015). In a more detailed study, Mandel’s team showed the inability of wild-type neurons to grow fine in the presence of *Mecp2* null astrocytes and proposed that, in RTT females, the activity of healthy neurons might be affected by *MECP2*-mutant astrocytes in a non-cell autonomous manner, probably through the impaired release of morphogenic factor(s) or/and secretion of toxic molecule(s) (Ballas et al., 2009). Few years later, by using inducible Cre mouse lines, they selectively removed or induced *Mecp2* in astrocytes and proved that while neurons are mainly responsible for the neurological phenotypes in RTT, astrocytes largely contribute to the progression of the disease (Lioy et al., 2011).

Emerging evidence has also reported a possible involvement of microglia and oligodendrocytes in RTT pathogenesis (Maezawa and Jin, 2010; Nguyen et al., 2013; Kahanovitch et al., 2019).

## Mouse models of *MECP2*-related disorders

Given their large-scale utility, several mouse models of RTT and *MECP2*-related disorders have been generated to investigate MeCP2 functions and the mechanisms underlying disease pathology (Ricceri et al., 2008; Lombardi et al., 2015). The first two *Mecp2*-null mouse models, generated with the Cre-Lox technology in the early 2000s, were developed in the laboratories of Rudolf Jaenisch and Adrian Bird (Chen et al., 2001; Guy et al., 2001). Both models carried deletion of exon 3 (Jaenisch) or exons 3–4 (Bird) of *MECP2* gene and well recapitulate many RTT features, thus further providing the genetic cause of the disease (Figure 1C). In particular, *Mecp2* null males (*Mecp2*^−/y^) have no apparent phenotype until 4 to 5 weeks of age, when they become underweight and exhibit hindlimb clasping, abnormal gait, tremors, breathing irregularities, and often seizures. Symptoms worsen with aging and the animals die approximately in 10–12 weeks. Compared to wild-type animals, null mice have smaller brains, shrinked cortices, more densely and packed neurons with immature synapses. Heterozygous female mice (HET, *Mecp2*^−/+^) display similar RTT-like phenotypes, including hypoactivity, ataxic gait, hindlimb clasping, breathing irregularities starting much later, at 3–4 months of age. In contrast to null males, they are fertile, become overweight and survive longer than 10 months. Although HET females should be the appropriate genetic mouse model of RTT, the long time required for symptoms to become overt and the associated phenotypic variability, led researchers to set their experiments on the *Mecp2* null male model, which manifests earlier and highly consistent phenotypes. However, the majority of RTT patients harbors missense or truncating mutations leading to a hypofunctional MeCP2 rather than to its complete loss. Accordingly, missense variations such as R106W, T158M, p.Thr158Ala (T158A) and p.Tyr120Asp (Y120D) express reduced protein levels compared to the wild-type product (Goffin et al., 2012; Johnson et al., 2017; Lamonica et al., 2017; Gandaglia et al., 2019). Interestingly, among nonsense mutations, the R294X produced stable truncated proteins whereas the R168X, R255X, and R270X did not yield to a detectable product (Collins and Neul, 2022). It is subject of debate whether global MeCP2 deficiency correctly recapitulates the molecular features of the disease. For this reason, other models harboring common *MECP2* mutations have been generated offering a long list of disease modeling animals for the comprehension of molecular consequences, pathophysiology and genotype–phenotype correlations of specific genetic lesions (Katz et al., 2012). The first RTT mouse model (*Mecp2*^308/y^) expressing a hypomorphic truncated form of Mecp2 and lacking of the C-terminal domain, was developed in 2002 (Shahbazian et al., 2002) (Figure 1C). The overall phenotype of the animal was milder with respect to the full null line; heterozygous females confirmed to be less sick and to manifest more variable phenotypes. Mice carrying truncating mutations (i.e., Mecp2 R168X or R255X), or mimicking the most common missense variations (i.e., Mecp2 p.Ala140Val (A140V), R133C, R306C, T158A/M, R106W) (Jentarra et al., 2010; Goffin et al., 2012; Lyst et al., 2013; Johnson et al., 2017; Lamonica et al., 2017) or the rare one Y120D (Gandaglia et al., 2019) have been generated, although the mostly used RTT mouse models still remain the *Mecp2*^−/y^ and *Mecp2*^308/y^. To better investigate the circuits involved in RTT pathogenesis and understand the etiology of the disease, conditional knockout mice have been developed and characterized. For instance, to achieve loss of *Mecp2* in cell types such as neurons and glia, a Nestin-driven Cre recombinase mouse line was used. The resulting animals showed reduced Mecp2 expression from embryonic day 12 (E12) and phenotypes similar to null mice, thus suggesting that the absence of Mecp2 in CNS is the leading cause of RTT symptoms (Chen et al., 2001). Subsequent studies addressed the consequences of *Mecp2* inactivation in specific neuronal subtypes or brain areas. For example, loss of Mecp2 in dopaminergic neurons caused impairment of motor coordination, while its absence in serotoninergic neurons induced augmented aggression (Samaco et al., 2009). Similarly, deletion of *Mecp2* in the basolateral amygdala resulted in anxiety behavior and learning deficits, while its loss in hypothalamic Sim1-expressing neurons revealed a role of Mecp2 in the regulation of social and feeding behavior and response to stress (Fyffe et al., 2008). Overall, each mouse line recapitulated some of the typical RTT features suggesting that MeCP2 function is important across brain regions, that all together contribute to the RTT features seen in patients. Conditional mice have been instrumental also to assess the neuropathological consequences of postnatal inactivation of *Mecp2.* Indeed, depletion of *Mecp2* at different ages (3 weeks-old and 10 weeks-old in males and 20 weeks-old in females) always caused the appearance of RTT-like phenotypes, brain shrinking and premature death (McGraw et al., 2011; Cheval et al., 2012; Nguyen et al., 2012). Although *Mecp2*-inactivating mutations have strong consequences on brain functioning, they do not lead to neuronal loss (Akbarian, 2003). Accordingly, a breakthrough study in 2007 demonstrated that reactivation of *Mecp2* in adult male and female mutant mice rescued neurological defects. These experiments proved that RTT and *MECP2*-related disorders are not an irreversible condition and that therapies focused on MeCP2 restoring could be beneficial in patients also after symptoms onset (Guy et al., 2007). Finally, mice overexpressing *Mecp2* (Mecp2-TG1) have been generated to investigate the gain of function consequences of the protein. Similarly to human pathology, severity of MDS-like phenotypes positively correlates with the levels of Mecp2 protein. Animals that overexpress modest level of Mecp2 showed enhanced motor learning, forepaw clasping, and increased contextual fear conditioning (Collins et al., 2004). In contrast, mice expressing from two to four-fold levels of Mecp2 displayed enhanced anxiety-like behavior and motor dysfunction. To conclude, mouse models represent the species mainly used to understand specific aspects of RTT pathology and to address preclinical studies of the disease; however other animal models such as rats, zebrafish and non-human primates have been developed and are available for RTT community to corroborate scientific findings (Veeraragavan et al., 2016; Chen et al., 2017).

## MeCP2: a multifunctional protein inducing a plethora of possible pathogenic mechanisms

**Figure 2:**
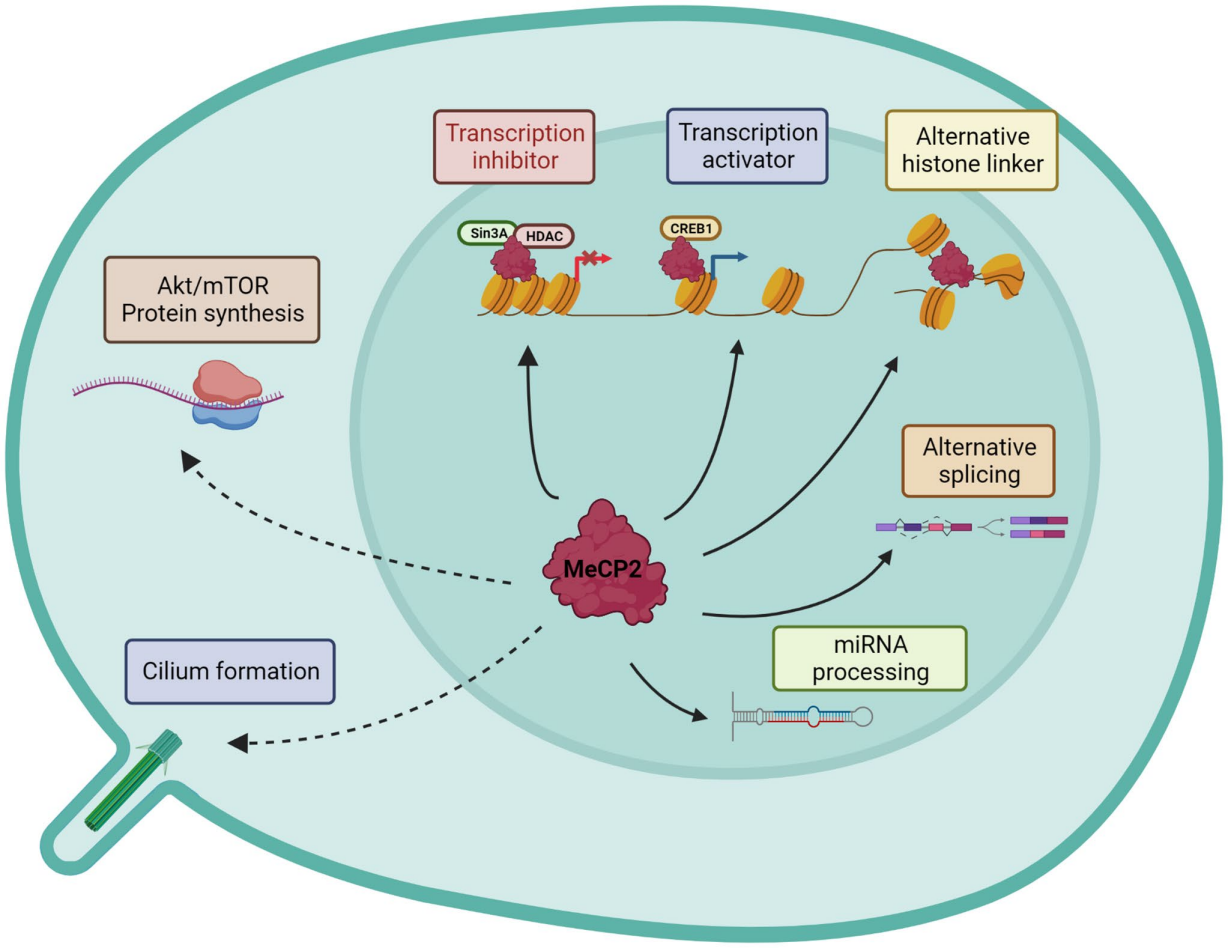
Graphical representation of known MeCP2 functions. Nuclear and cytoplasmatic activities are distinguished. Continuous lines describe direct MeCP2 actions, while dotted lines the indirect effects.

By the time that *MECP2* was identified as a causative gene of RTT, Adrian Bird and his collaborators had already partially characterized its functions (Lewis et al., 1992). Indeed, their studies had shown that MeCP2 selectively binds to methylated DNA independently from the specific sequences, thereby repressing transcription *in vitro*. On the same line, it was proved that the TRD domain promotes gene silencing by binding to corepressor complexes (Sin3A and NCoR) that contain histone deacetylase activities (Jones et al., 1998; Nan et al., 1998) (Figure 2). Subsequently, Skene and colleagues proposed that in mature neurons, Mecp2 might serve as an alternative linker histone and organize a specialized chromatin structure, thus dampening overall transcriptional noise (Skene et al., 2010). In addition, MeCP2 function was linked to mRNA splicing for its interaction with Y-box binding protein 1 (YB-1), a member of the family of DNA- and RNA-binding proteins, implicated in many RNA/DNA dependent processes including the regulation of alternative splicing (Eliseeva et al., 2011). Since then, many other splicing factors have been associated with MeCP2, especially through its CTD or TRD domains (Good et al., 2021), leading Cheng and colleagues to propose that the majority of MeCP2-bound proteins are involved in RNA splicing and processing (Cheng et al., 2017). However, few years later Chhatbar and co-workers demonstrated that regulation of splicing is not a primary function of MeCP2 (Chhatbar et al., 2020). To increase the repertoire of roles attributed to MeCP2, other reports have proposed for MeCP2 a regulatory function in microRNA (miRNA) post-transcriptional processing (Cheng et al., 2014). Moreover, contrary to expectation, *ex-vivo* studies on purified hypothalami and cerebella from RTT mice suggested that MeCP2 may also positively regulate gene expression by interacting with the transcriptional activator cAMP response element-binding (CREB) (Chahrour et al., 2008). In 2011, the protein synthesis was found significantly impaired in both *Mecp2* null mice and heterozygous females as a consequence of reduced AKT/mTOR signaling pathway (Ricciardi et al., 2011), thus suggesting yet another function of MeCP2. However, this study did not address how the translational deficits contribute to RTT pathogenesis, nor indicated whether they are a direct or indirect effect of Mecp2 loss. Finally, we recently published that MeCP2 localizes at the centrosome and in primary cilium thus providing another role for the protein outside the nucleus (Bergo et al., 2015; Frasca et al., 2020). Interestingly, we showed that *Mecp2* loss affects cilium formation and signaling transduction of the Sonic Hedgehog pathway, a key regulator of processes involved in brain development and growth (Frasca et al., 2020) (Figure 2). All together, these findings emphasize the multifunctional role of MeCP2 in brain, even though its main role appears to be its capacity to bridge the NCoR1/2 corepressor complex to methylated DNA (Tillotson and Bird, 2019). Although all these studies suggest a primary role for MeCP2 in regulating gene expression, it is important to mention that Johnson et al. (2017) have recently proven that most of the transcriptional changes observed in *Mecp2* deficient neurons are cell type-specific, therefore reflecting the high cellular heterogeneity featured by brain. Further, by analyzing, in *Mecp2* heterozygous female mice, gene expression profiles of neurons expressing either the wild type or mutant *Mecp2* allele, they revealed that both cells feature differentially expressed genes (DEGs). Interestingly, most DEGs occur in neurons expressing the mutant allele, thereby indicating cell autonomous changes; however, non-cell autonomous DEGs were also reported. Notably, cell and non-cell autonomous DEGs represent different biological processes (Johnson et al., 2017).

## Genetic approaches targeting the *MECP2* gene or its transcript

**Figure 3:**
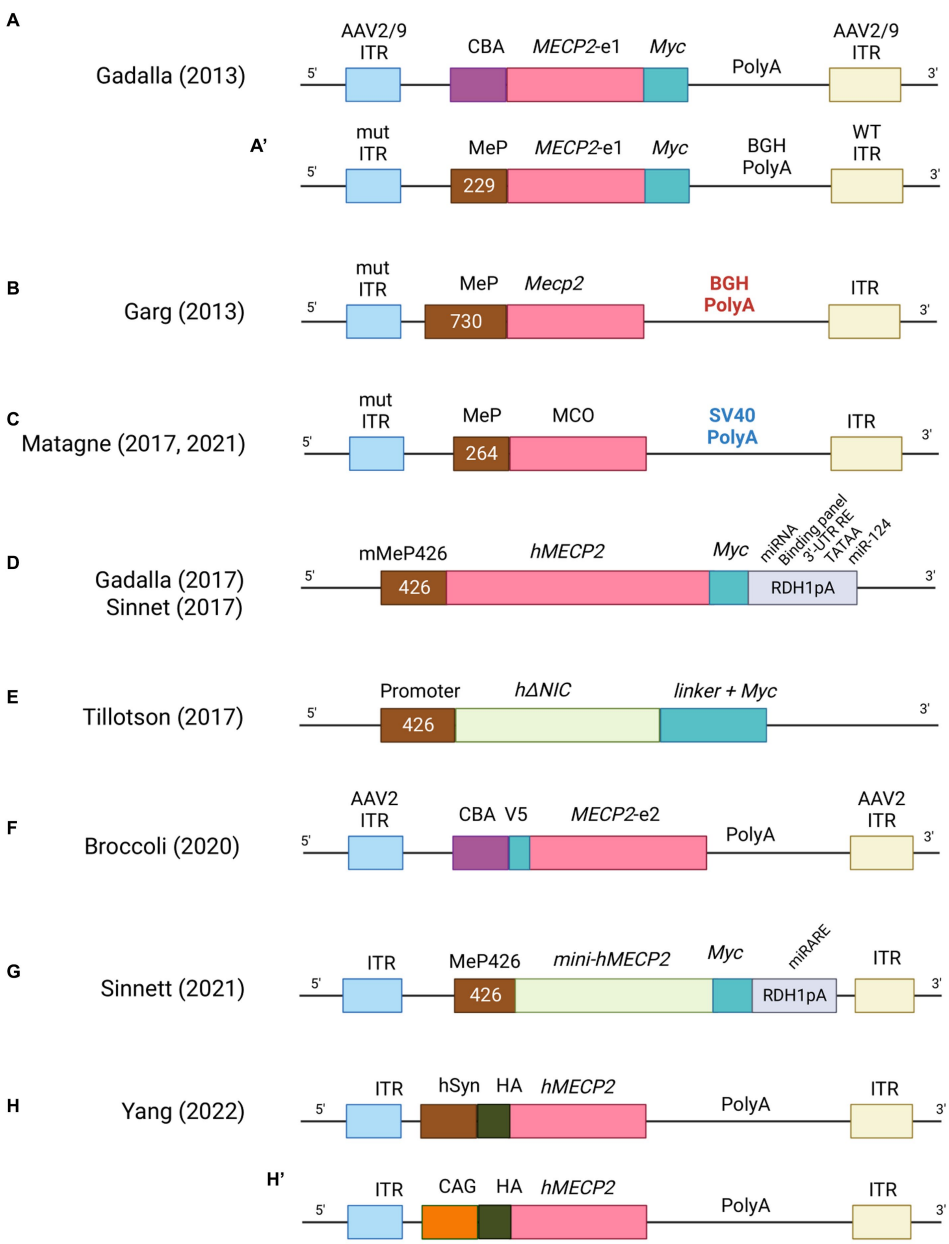
List of AAV constructs used in gene therapy for RTT. For each construct the main features are indicated. CBA = Chicken β-actin promoter, MeP = MeCP2 promoter, ITR = Inverted terminal repeats, BGHpolyA = bovine growth hormone polyadenylation signal, SV40polyA = Simian virus 40 hormone polyadenylation signal, hSyn = human synapsin 1 gene promoter, CAG = cytomegalovirus early enhancer/chicken β-actin promoter.

Since the ground-breaking idea of using viruses as vector for gene therapy, much progress has been made to develop an efficient methodology. Retroviruses, lentiviruses, adenoviruses and adenovirus-associated viruses (AAV) have been widely studied to deliver therapeutic genes in diverse applications and in the past decade to treat multiple disorders (Bulcha et al., 2021). In particular, lentiviral and retroviral vectors were the earlier employed to treat disease models (Miller, 1992). However, their big advantage of carrying larger DNA payload was mitigated by the fact that the integration of fragment of their genetic material into the genome of the host cell increased the probability of insertional mutagenesis and carcinogenesis (McCormack and Rabbitts, 2004; Hacein-Bey-Abina et al., 2008). In addition, these viruses do not cross the blood brain barrier (BBB), thus resulting less appropriate for clinical trials of neurological disorders. In contrast, adenoviruses and AAVs can efficiently bypass the BBB, infect post-mitotic cells such as neurons, and provide stable transgene expression without integrating within the host genome (Foust and Kaspar, 2009). Unfortunately, adenoviruses induced an elevated immune response from the target cells which limited their use in clinics. Remarkably, AAVs triggered very low levels of immune-response and for this reason were the first to be used in clinical trial in 2012 to treat lipoprotein lipase deficiency, a rare autosomal recessive disorder of lipid metabolism (Carpentier et al., 2012). Since then, several clinical trial for CNS disorders (Sanfilippo type A and B, ClinicalTrials.gov: NCT02053064 and NCT03300453, respectively; Batten disease, ClinicalTrials.gov: NCT01414985; metachromatic leukodystrophy, ClinicalTrials.gov: NCT01801709; and spinal muscular atrophy, ClinicalTrials.gov: NCT02122952) have utilized AAVs as gene delivery tool (Gray et al., 2011b; Saraiva et al., 2016). However, one limitation of these vectors is their small packaging size (~5.0 kb) which restricts the product that can be packed to less than 4.7 kb for single stranded (ss) and approximately to 2.3 kb in the more efficacious self-complementary (sc) packaging approach (McCarty et al., 2001; Lykken et al., 2018). Currently, two methods have been employed to deliver these vectors into the brain: systemic (e.g., intravenous injection) and direct CNS route (e.g., intrathecal, intra-cisterna magna or direct injection into the neuropil). Systemic delivery is more desirable from a translational point of view because it is less invasive than the direct routes, although the number of transduced cells in the CNS is limited and the choice of AAVs vector able to cross the BBB is restricted to serotype 9 (AAV9) (Gray et al., 2011b; Saraiva et al., 2016) as demonstrated in mice and large animal models (Foust et al., 2009; Bevan et al., 2011; Mendell et al., 2017). The translational feasibility of systemic administration of AAV has a big limitation though: the high doses required to efficiently transduce the CNS and promote diffuse expression of the transgene often lead to off-target-based toxicity, especially in the liver (Ronzitti et al., 2020; Verdera et al., 2020). To improve tissue tropism and avoid systemic immune responses, AAV viral genome has been widely engineered with the inclusion of cell-type specific promoters (Gray et al., 2011a; de Leeuw et al., 2014), enhancers elements (Vormstein-Schneider et al., 2020; Graybuck et al., 2021), microRNA target sites (Hordeaux et al., 2020) and viral capsid variants (Matsuzaki et al., 2018; Ravindra Kumar et al., 2020). In the next paragraph, we will describe the evolution of AAVs constructs (Figure 3) and their pros and cons in pre-clinical studies of RTT (Supplementary Table S1).

## Gene replacement therapy in RTT

Given that RTT is a monogenic disorder caused by loss of function mutations in *MECP2* and that its protein has crucial roles in several physiological pathways, gene delivery of a wild copy of *MECP2* to mutant brain cells have been widely contemplated for the treatment of RTT. A seminal study by Rastegar and colleagues demonstrated the capacity of retroviral vectors comprised of self-inactivating (SIN) long term repeat (LTR) regions and of the *Mecp2* promoter (MeP) to efficiently transduce *MECP2-e1* in neuronal stem cells (NSCs). Differentiated neurons derived by infected NSCs showed restoration of protein levels and rescue of dendritic growth and branching. Consequently, the authors employed SIN lentiviral vectors to directly transduce mature cortical neurons. However, lentiviral vectors only reached a restricted brain area near to the administration site, indicating that they are probably unsuitable for the treatment of RTT (Rastegar et al., 2009).

### 1st generation of expression cassettes

The first and encouraging report of gene therapy in mouse model of RTT was obtained by administrating recombinant adeno associated viruses, that still appears as the preferred vectors. In particular, brain injection of single stranded AAV2/9-*MECP2,* driven by the chicken β-actin promoter (AAV2/9-CBA-*MECP2;* Figure 3A) in postnatal day (P) *Mecp2*-null mice (P0-2), efficiently transduced different areas (almost 40% in thalamus and hypothalamus, 20–25% in motor cortex and hippocampus, 15% in brain stem and only 7% in the striatum) (Gadalla et al., 2013). Transgene expression was found mostly in neurons and at near physiological levels. Infection efficiency was sufficient to ameliorate some RTT-like phenotypes, such as motor functions and life span. In the same study, systemic administration in juvenile mice (4- to 5-weeks old) of a vector (scAAV9-MeP-*MECP2;* Figure 3A) in which *MECP2* expression was guided by a short region of its own promoter (229 bp), although drastically reducing transduction efficiency in brain cells (2–4%), was able to extend life span (Gadalla et al., 2013). Studies of biodistribution revealed higher transduction efficiency in peripheral tissues, mostly in liver. Notably, high levels of alanine aminotransferase, a liver damage marker, were observed in treated animals, suggesting that MeCP2 overexpression leads to deleterious side effects. Parallelly, systemic delivery in RTT male mice of 4- to 5-weeks of a vector containing the *Mecp2*e1 cDNA guided by a longer fragment of its own promoter (~700 bp; Figure 3B) robustly improved RTT features and stabilized phenotypes (i.e., survival and phenotypic score). The good transduction efficiency in CA3, brain stem and cortex (20–25%) could explain the observed stabilization, while the lower efficacy in cerebellum (5%) might indicate the minor importance of reaching this area (Garg et al., 2013). Accordingly, a less prominent role for cerebellum in the progression of RTT mouse phenotype has been recently suggested (Achilly et al., 2021; Carli et al., 2023). Notably, systemic injection in 10- to 12- months old heterozygous (HET) females also improved phenotypic score and motor function while the effects on respiration were inconclusive and liver damage was not observed (Garg et al., 2013). In addition, Roux’s team demonstrated that systemic injection in 5 months-old *Mecp2* HET female mice of the sc-AAV9-MCO vector, expressing the codon-optimized *Mecp2e1* (MCO) transgene controlled by a short portion of the *Mecp2* promoter (264 bp; Figure 3C), rescued breathing deficits and improved locomotor functions (Matagne et al., 2021). Administration of the same virus to *Mecp2*-null mice improved motor functions, explorative behaviors and extended the median life span; respiratory patterns were also normalized (Matagne et al., 2017). All together these studies highlighted that an efficacy of brain cells transduction of 25–40% is sufficient to ameliorate mouse phenotypes, but it could not rescue RTT-like features to wild-type levels which probably requires a higher transduction efficiency. Moreover, it raised a concern about the toxic consequences induced in peripheral tissues by systemic delivery of the MeCP2 therapeutic gene. More recently, the endogenous *Mecp2* promoter was replaced with the human synapsin (hSyn) (Figure 3H) or the cytomegalovirus early enhancer/chicken β-actin (CAG) promoter (Figure 3H) (Yang et al., 2023). Similar to previous reports, brain injections into the later ventricle (LV) of P2 *Mecp2^−/y^* mice resulted in higher transduction efficiency for hypothalamus and lower for hippocampus and cortex. Interestingly, when mice were injected with AAV9-CAG-*MECP2,* MeCP2 levels increased less (38,9%) compared to the AAV-hSyn-*MECP2* vector, but the median survival was extraordinary prolonged, possibly because of the capacity of the AAV9-CAG-*MECP2* vector to restore MeCP2 expression in astrocytes and oligodendrocytes (Yang et al., 2023). None of these vectors raised liver toxicity.

### 2nd generation of expression cassettes

With the aim to control the levels of MeCP2 and deliver the therapeutic gene more efficiently without toxicity, a second generation of expression cassette was investigated (Gadalla et al., 2017; Sinnett et al., 2017). This new AAV9 vector, in addition to the *MECP2e1* cDNA controlled by its own promoter (MeP426), included a modified 3′ UTR incorporating its highly conserved polyadenylation (pA) signal, and a panel of miRNA-binding sites relevant to control *MECP2* mRNA levels (Figure 3D). Intravenous delivery of the generated virus in juvenile mice (4- to 5-weeks old) did not affect hepatic architecture despite its higher transduction and, similarly to the first generation of AAV9, improved life span and body weight of mutant animals while it did not ameliorate the severity score. In contrast, direct intracerebroventricular (ICV) injection into neonatal *Mecp2-*null mice resulted in higher brain transduction efficiency (comparable to that one described in Gadalla et al., 2013), increased survival and ameliorated RTT-like phenotypes, outlining the importance of endogenous regulatory elements and the need of high transduction efficiency throughout the brain (Gadalla et al., 2017). The same 2nd generation vector was injected into the cisterna magna of juvenile *Mecp2* mutant mice (Sinnett et al., 2017) (Figure 3D). Although treated animals improved lifespan and body weight, behavioral traits were not rescued in contrast with previous reports. To further ameliorate the AAVs strategy, novel capsid variants were engineered. A recent work used the synthetic vector, AAV-PHP.B which features higher permeabilization of the BBB in adult mice and more efficient transduction of neurons and glia (Deverman et al., 2016; Morabito et al., 2017) (Figure 3F). An instable *Mecp2* (i*Mecp2*) transgene cassette was then inserted to limit supra-physiological levels of Mecp2. The intravenous injection of the PHP.eB-iMecp2 vector in 4 to 5 weeks old male mice revealed a sustained behavioral improvement when at least 70% of the brain cells were infected and physiological levels of the protein were maintained (Luoni et al., 2020). Importantly, an efficiency of 15% of brain cells transduction was not sufficient to ameliorate RTT-like phenotype; further, infected *Mecp2* null male mice displayed a strong immune response to the exogenous protein which severely affected their lifespan. To overcome this issue, chronic immunosuppression was employed leading to strikingly ameliorated general health conditions and prolonged life span. Systemic delivery of the PHP.eB-iMecp2 vector in 5 months old *Mecp2^+/−^* HET females improved locomotor phenotypes and pathological features; no hepatotoxicity was observed (Luoni et al., 2020). Although these findings reinforced the idea that gene therapy could be a promising strategy for RTT, it has to be noticed that the brain tropism of this vector is unfortunately restricted to C57Bl/6 J mice and the LY6A receptor mediating its efficient transport through the BBB is not expressed in non-human primates (NHPs) (Hordeaux et al., 2018).

The most successful study based on engineered capsid variants was recently described by the Gradinaru’s lab. By applying the Multiplex-Cre recombination-based AAV targeted evolution (M-CREATE) method (Ravindra Kumar et al., 2020; Goertsen et al., 2022), the authors identified the variant AAV.CAP-B10 which showed higher tropism for neurons and negligible specificity for all peripheral tissues including liver. Intravenous administration of this vector in mice and adult marmosets resulted in broad and robust transgene expression across cortex and cerebellum as well as spinal column and dorsal root ganglia (DRG) regions (Goertsen et al., 2022). On the contrary, the delivery of AAV-PHP.B failed to increase transgene expression in the brain of marmosets confirming previous data in NHP (Matsuzaki et al., 2018). In general, this study posed an important step forward for the treatment of neurological disorders by gene therapy.

## Mini*MECP2* expression cassettes

To increase the efficiency of transduction, several laboratories focused on scAAVs; the reduced packaging capacity (2.2 kb) (McCarty et al., 2001) led the Bird’s team to include only essential domains of the transgene. Interestingly, intracranial injection in neonatal mice of vectors encoding a minimal-MeCP2 protein (scAAV-mini *MECP2*) constituted by the methyl-binding domain and the NCOR- interaction domain (NID) improved phenotypes and survival in the absence of toxic effects (Tillotson et al., 2017) (Figure 3E). As logic consequence, Sinnet and colleagues paired the strategy of inserting microRNA targets into the 3’ UTR of *MECP2* with scAAV9 vector carrying mini*MECP2*. In particular, they designed a novel miRNA target panel (named miR-responsive autoregulatory element or miRARE) able to “tune” mini*MECP*2 expression through a negative feedback mechanism that is responsive to *MECP2* overexpression (Sinnett et al., 2021) (Figure 3G). This work showed that the inclusion of the autoregulatory element improved the safety of AAV9/mini*MECP2* gene therapy without compromising its efficacy (Sinnett et al., 2021). Moreover, biodistribution analysis revealed that while miRARE inhibited the expression of MeCP2 in wild-type mice (~8% of brain cells expressed transduced MeCP2), in mutant animals, 40% of brain cells expressed the therapeutic gene. Importantly, injected null mice showed delayed onset of gait abnormalities and extended life span.

Although gene delivery is becoming a promising strategy for several neurological disease, an important concern for developmental disorders is to define the optimal time-window of intervention. Published data from preclinical studies of RTT gene therapy indicated that delivery of *MECP2* either in newborn pre-symptomatic or symptomatic adult mice can ameliorate survival and recover phenotypes, thus suggesting that therapeutic interventions can be potential effective across different ages. However, a systematic study exploring different time windows of MeCP2 delivery has not been performed yet. Regardless of the time of intervention, the long-lasting expression of transgene at appropriate therapeutic dosage is of primary importance to avoid repetitive treatments that are not always accessible especially in case of neurological disorders. Indeed, to the best of our knowledge, no study has reported how long the *MECP2* transgene remains expressed or when it turns off. Further, we find it relevant to disclose which is the right number of brain cells that have to be transduced for significant clinical improvement and if specific brain areas have to be primarily transduced; further, although intuitive, the importance of expressing the *MECP2* transgene also in glial cells remain to be proven. On this line, considering that: i) gene replacement occurs both in cells expressing the WT or the mutant allele and with uneven doses and ii) *MECP2* dosage appears to affect gene expression with cell and non-cell autonomous effects (Johnson et al., 2017), in the future, it might be highly informative to assess at the cellular level the transcriptional consequences of gene therapy. In light of that, AAV9 vectors carrying different promoters may be chosen to preferentially target specific cell types of the brain.

## *MECP2* gene and RNA editing

**Figure 4:**
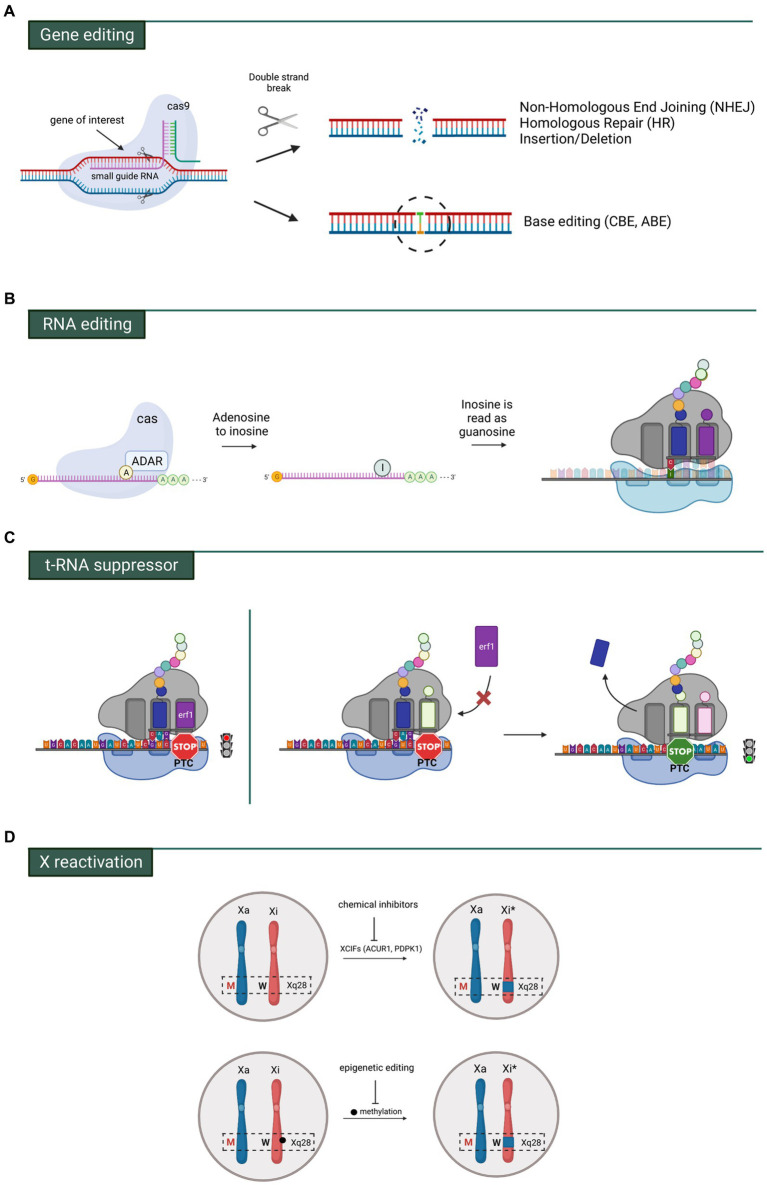
Main genetic strategies investigated for RTT. **(A)** Gene editing by means of Crisp-CaS9 allowing broad changes in portions of DNA or precise single base modifications. **(B)** RNA editing comprising the ADAR system which allows to modify an adenosine with inosine through deamination. Inosine is then recognized as a guanosine and paired with a cytosine in the anticodon tRNA. **(C)** On the left, a prematur termination codon (PTC) interrupts the translation of the mRNA. On the right, a specific tRNA suppressor binds the PTC allowing the translation readthrough. Erf1 = eukaryotic translation terminator factor 1. **(D)** The mutated *MECP2* allele (red M) is localized in the active X-chromosome (Xa), while the wild type *MECP2* allele (black W) is in the inactive chromosome (Xi). By means of chemical (silencing of inactivating factors) or epigenetic actions (inhibiting DNA methylation), the region containing the wt allele of *MECP2* (Xi*) can be reactivated.

A valid alternative strategy to maintain correct MeCP2 levels and avoid overexpression side effects is represented by genome editing which directly repairs the mutated gene. The most popular method of gene editing is CRISPR-Cas9 composed by the Cas9 enzyme, a nuclease capable of cutting the genome at predefined location indicated by a guide RNA (gRNA) and a repair template, which contains a wild-type sequence. These two components form a ribonucleic complex that recognizes and cleaves the target sequence (Figure 4A). Depending on cell type and its growth phase, cleaved DNA can be repaired either by non-homologous end joining (NHEJ) or homology-based repair (HDR) (Ran et al., 2013; Jiang and Doudna, 2017). HDR requires long homology arms to precisely introduce the exogenous DNA template (ssDNA or dsDNA) and finely edit the break site (Wu et al., 2018). Its action is favored during S, G2 and M phases, but it is strongly repressed in G1. NHEJ repairs the double strand breaks (DSBs) during all cell cycle phases, but its highest activity has been found in G1. For these reasons NHEJ-based methods have been mostly employed for genome editing in neurons, although they usually generate small insertions/deletions (indels) around the break site. Notably, HDR has been recently demonstrated to be effective in terminally differentiated neurons, although at lower levels (Nishiyama et al., 2017). However, one of the main caveats that limits the use of the CRISPR-Cas9 system in gene therapy is the necessity of co-transducing in the same cell two different AAVs, respectively containing the Cas9/gRNA and the “template.” Another concern is their long-term presence which may lead to off-target cleavage mediated by the Cas9 enzyme or to alteration of relevant and “dangerous” genes (Zhang et al., 2015). In spite of that, because of its high efficiency and feasibility, CRISPR-Cas9 approaches are nowadays leading research studies for the treatment of several genetic diseases. Regarding RTT, the first *in vitro* study was conducted into human induced pluripotent stem (iPS) cells (le et al., 2019). By co-transfecting the sg5/Cas9 and the ssODN-R270X template vectors, the MECP2^R270X^ mutation was initially knocked-in leading to two homozygous and five heterozygous clones out of 22, thus reaching an insertion efficiency of 9 and 23%, respectively. With the focus to correct the variation, homozygous iPSC clones (*MECP2*^R270X/R270X^) were then transfected with sg3/Cas9 and donor wild type template vectors. Consequently, sequencing analysis revealed the successful repair of mutant *MECP2* in iPSCs and the recovery of its mRNA levels (le et al., 2019). Another encouraging study of genome editing in RTT was performed in patient fibroblasts and neurons derived from iPSCs carrying the most common *MECP2* variant, c.473C > T p.Thr158Met (Croci et al., 2020). Co-transfection efficiency of the dual vector approach was about 8,6% in RTT fibroblasts. Next generation sequencing of sorted cells harboring both vectors revealed that on average 55% of alleles were correctly edited, leading to a significant increase of MeCP2 levels. Similarly, 14% of mutant alleles were reverted to the WT sequence in iPSC-derived neurons (Croci et al., 2020). The lower editing efficiency in neuronal cells was probably due to the aforementioned HDR deficiency in post-mitotic cells (Chapman et al., 2012; Bonnerjee and Bagh, 2021). Although these *in vitro* data represented a first step towards gene therapy approaches based on the application of CRISPR/Cas9 system, to date no study has ever tested this tool in animal models of RTT.

To circumvent the generation of DSB and reduce indel modifications, a DNA base editing tool has been recently developed that enables a direct and irreversible conversion of one base pair to another at the target site (Gaudelli et al., 2017). This strategy is based on the fusion of Cas9 mutants, that cannot make DSBs, with specific nucleotide-converting enzymes named editors. Two different base switching are nowadays possible: C-G to T-A mediated by cytosine base editors (CBEs) and A-T to G-C catalyzed by adenosine base editors (ABEs) (Gaudelli et al., 2017). However, the only base-editing strategy so far tested in *in vivo* model of RTT is the RNA Editing for Programmable A to I Replacement (REPAIR). This approach takes advantage of a family of naturally occurring enzymes named Adenosine Deaminase Acting on RNA (or ADAR) (Maas et al., 1996; Melcher et al., 1996; O’Connell et al., 1998), which catalyze the hydrolytic deamination of adenosine (A) to inosine (I) on RNA (Bass and Weintraub, 1988). The inosine present on the mRNA codon pairs with a cytosine (C) present on the tRNA anticodon, therefore resulting in codon change (Figure 4B). One member of ADAR family, ADAR2, is highly expressed in brain and mostly engineered for site-direct RNA editing. Its domains have been fused with heterologous RNA binding proteins (i.e., Cas13 or the bacteriophage λN peptide) to improve the capability to specifically target endogenous RNA by recognizing short hairpin RNA (shRNA). Indeed, ADAR2 binds to the shRNA that is complementary to the mRNA of interest and deaminases the target sequence (Cox et al., 2017). Considering that approximately 55% of pathogenic variants causing RTT are G > A changes, repair by targeted RNA editing represents a valid alternative approach (Fyfe et al., 2003). With this purpose, the laboratory of Gail Mandel generated a Knock-in (KI) mouse carrying the c.317G > A, p.Arg106Gln (R106Q) mutation in the *Mecp2* gene (Sinnamon et al., 2017). Infection of cultured hippocampal neurons with AAVs containing a hyperactive ADAR2 enzyme fused to a bacteriophage peptide and a guide shRNA sequence, resulted in *in vitro* RNA repair and the recovery of Mecp2 protein level (about 40%) together with its ability to bind to heterochromatin (Sinnamon et al., 2017). Few years later, the same group tested the efficacy of RNA editing in a mouse model of RTT. AAVs expressing the RNA editing enzyme were injected into the *Mecp2* null hippocampus and, 3 weeks later, 50% of *Mecp2* mRNA was edited (i.e DG, CA1 and CA3) (Sinnamon et al., 2020). Very recently, the same authors provided the first evidence that targeted RNA-editing approach can alleviate behavioral phenotypes in a mouse model carrying a human mutation. To assess a different adenosine context, they generated a novel RTT mouse model carrying the patient mutation *MECP2^G311A^* (Sinnamon et al., 2022). Systemic injection of viral vectors into retro-orbital region of P28 and P35 adult animals showed the highest efficiency of RNA-editing in the brainstem (18%) and midbrain (13%) after 4 weeks from the delivery. The other brain areas such as cerebellum, cortex, olfactory bulb, thalamus, hypothalamus and striatum only reached 3–5% of efficiency. In particular, MeCP2 protein expression was restored in 20% of brainstem cell populations where its association with heterochromatic foci was almost fully rescued (75% of Mecp2 intensity in heterochromatic foci). The half lifespan of injected mice with *Mecp2*-targeting virus was extended of about 6 weeks compared to control animals. Moreover, since the brainstem is a brain region tightly linked to respiratory function, the authors mainly focused on the evaluation of apneas and breathing patterns, which were improved in treated animals, while motor or cognitive functions were not tested (Sinnamon et al., 2022).

Although further *in vivo* studies are necessary to confirm its therapeutic efficacy, RNA editing could represent, in near future, the approach of choice for patients with suitable mutations.

## Readthrough of *MECP2* nonsense codon mutations and t-RNA suppressor therapy

Nonsense mutations are responsible for 10–15% of all genetic lesions and for almost 1,000 deleterious genetic disorders (Mort et al., 2008). Nearly 35% of typical RTT patients harbor *MECP2* nonsense mutations which lead to early protein truncation (Lyst and Bird, 2015; Ip et al., 2018). A very attractive pharmacogenetic strategy that mediates the suppression of these mutations by small molecules is provided by translational readthrough inducing drugs (TRIDs) (Figure 4C). Basically, aminoglycosides, such as gentamicin, can restore the expression of full-length proteins by allowing readthrough of premature termination codons (PTCs). Mechanistically, gentamycin weakly binds to the eukaryotic ribosomal decoding center, leading to misincorporation of near-cognate aminoacyl-tRNAs at the PTC, therefore permitting to continue protein synthesis albeit inducing, in some cases, a missense mutation (a concerning issue considering that MeCP2 is particularly sensitive to residue alterations) (Wilhelm et al., 1978; Palmer et al., 1979). In any case, several studies have showed elevated toxicity for gentamycin at required therapeutic doses (Karijolich and Yu, 2014) and its restricted ability to cross the BBB (Nau et al., 2010). To overcome these limitations, novel-related compounds (including Ataluren (or PTC124), ELX-02, and NB54) were tested in animal models of human diseases, leading to successful clinical trials for Duchenne muscular dystrophy (DMD) and cystic fibrosis (CF) (Hamed, 2006). However, a phase 2 clinical trial of non-aminoglycoside drug, Ataluren, was recently showed to be not effective for the treatment of nonsense mutations in CDKL5 deficiency disorder (CDD) and Dravet syndrome (DS) (Devinsky et al., 2021). Accordingly, *in vitro* studies of Landsberger’s team demonstrated the inability of this drug to induce read-through activity on CDKL5 PTCs. Moreover, they also reported that conversely, aminoglycosides efficiently suppressed CDKL5 nonsense mutations and partially recovered the protein activity (Fazzari et al., 2019). Similarly, aminoglycosides administration in cultured cells overexpressing several common nonsense *MECP2* mutations, or cultured fibroblasts derived from RTT patients induced the expression of MeCP2 that correctly localized into the nucleus (Brendel et al., 2011; Vecsler et al., 2011). Interestingly, only one publication demonstrated, in an animal model carrying the *Mecp2*-R294X PTC, the capacity of gentamicin to increase the expression of full-length Mecp2 (Merritt et al., 2020); however, whether this increase was sufficient to ameliorate RTT phenotypes has yet to be tested. In spite of that, readthrough therapy has several disadvantages including low efficiency, high toxicity, not specificity and nucleotide context-dependency, that need to be overcome to make this approach suitable in the next future. A valid alternative strategy could be represented by nonsense suppressor tRNA (sup-tRNA) approach, which makes use of anticodon-engineered tRNAs able to recognize the PTC but charged with correct amino acid to permit translational readthrough. The delivery of sup-tRNAs through AAVs would thus stabilize the RNA and rescue the expression of the full-length protein (Chang et al., 1979; Temple et al., 1982). This concept was introduced decades ago and very recently it was tested in a mouse model of human lysosomal storage disease, the mucopolysaccharidosis type I (MPS I) caused by α-L-iduronidase (IDUA) enzymatic activity deficiency (Wang et al., 2022). Systemic injection of an rAAV9.2 sup-tRNA^Tyr^ restored IDUA activity in liver and heart lysates up to 9.5 and 27% of WT level respectively, however no activity was recorded in brain. In contrast, unilateral intrahippocampal injection of rAAV9.2 sup-tRNA^Tyr^ restored IDUA activity to 10% in the injected hippocampus (Wang et al., 2022), suggesting that sup-tRNA^Tyr^ works in various tissues although its feasibility is still limited by the efficiency of gene delivery. Although this technology is still at early development, and no study is reported in RTT, we envisage that in future it will largely benefit from the already available advanced approaches of gene transduction in brain.

## Reactivation of the inactive X chromosome

Due to XCI, RTT girls are mosaics of cells expressing either normal or mutant *MECP2* (Takahashi et al., 2008). Reactivation of the wild type allele on the inactive X chromosome (Xi) represents a potential therapeutic approach for RTT. Because of XCI reversibility, several laboratories attempted to find molecules able to promote its reactivation, by targeting its inactivating factors (Figure 4D). One of the first study conducted by Green and his collaborators reported that pharmacologically targeting of X chromosome inactivation factors (XCIF) such as ACVR1 (Activin A receptor type I) and PDPK1 (Pyruvate Dehydrogenase Kinase 1) reactivated *Mecp2* in the nuclei of differentiated mouse ES cells (Bhatnagar et al., 2014)*. In vitro* combined inhibition of ACVR1 and PDPK1 effectors rescued morphological defects in RTT neurons (i.e., soma size and dendritic branches) upon *Mecp2* reactivation. *In vivo* intracerebral injection of the same combined treatment in *XistΔ:Mecp2/Xist:Mecp2-GFP* female mice, harboring the deletion of the *Xist* gene and expression of wild-type *Mecp2* in the active X chromosome, and *Mecp2* fused to GFP on the inactive one, resulted in reactivation of Xi-Mecp2-GFP in 30% of cells (Przanowski et al., 2018). By digging into the mechanisms of Xi reactivation, Lee’s lab used a combination of an inhibitor of DNA methylation (5-Aza) with an antisense oligonucleotide (ASO) directed against *Xist* RNA to activate the inactive X chromosome. After five days of treatment with Xist ASO + 5-Aza, mouse embryonic fibroblasts (MEFs) carrying the *Mecp2:luciferase* reporter showed an encouraging strong increment of *Mecp2:luciferase* levels (Carrette et al., 2018). Additionally, luminescence-based high-throughput screens on mouse fibroblasts carrying an inactive *MeCP2-luciferase* reporter identified two inhibitors (AG490 and Jaki) of the JAK/STAT pathway as XCI reactivating agents. This study revealed that reactivation is cell-type dependent. Indeed, while AG490 and 5-Aza reactivated *Mecp2* in mouse fibroblasts, only 5-Aza increased MeCP2 levels in a humanized Xi-containing cell line (THX88) (Lee et al., 2020). To date, the mixed modality approach represents a valid strategy for the treatment of X-linked disorders and encourages further screening for Xi-reactivating drugs. However, one concern could be the tissue/cell-type specificity and the toxicity at high doses, that together with reactivation of other X-linked genes could lead to deleterious effects. Alternatively, a targeted approach, consisting in epigenetic editing, was recently tested in RTT-like hESCs carrying on Xi a wild-type allele of *MECP2* and its methylated promoter, and on the active X a *MECP2* null allele produced by a GFP-polyA stop cassette after exon 3. Transduction of hESC derived neurons with dCas9-Tet1/sgRNA proved that demethylation of the *MECP2* promoter reactivated the wild type allele located on the Xi (82% of protein expression). Moreover, direct epigenome editing of neurons, carrying on Xi MeCP2 exon 3 fused with GFP and its methylated promoter and on Xa MeCP2 exon 3 fused with tdTomato, showed a moderate reactivation of MeCP2-GFP (17,7% of protein expression) without affecting the expression of other genes on both X chromosomes (Qian et al., 2023). Although precise DNA methylation editing of *MECP2* displayed encouraging results, further validation in *in vivo* animal models of RTT is essential for the translational value of this approach. For instance, the big size of the current editor (dCas9-Tet1) that have to be packed in a single AAV for *in vivo* delivery could represents a big challenge for the pre-clinical studies.

## Future direction for RTT therapy: nanoparticles’ delivery

An attractive alternative method for gene delivery might be represented by nanoparticles; because of their lower immunogenicity, higher genetic payload and moderate costs, it is an emerging field that is being tested for several neurological disorders and brain cancers. There are three categories of nanomaterials: (i) lipid-based, (ii) polymer-based, and (iii) inorganic nanoparticles (Ramamoorth and Narvekar, 2015). Lipid-based nanoparticles (LBNP) represent a valid vehicle from a safety perspective because of their biodegradability and low toxicity. Even though they are not much stable, they have been widely explored and already used in clinic for a range of diseases including cancer (Vaughan et al., 2020) and the recent COVID-19 vaccines (Andresen and Fenton, 2021). Polymer-based nanoparticles (PBNP) are more stable than LBPN and display controlled degradation and elimination of polymer (Moku et al., 2021). The main disadvantages are constituted by poor targeting and quick nanoparticles clearance (Gagliardi et al., 2021) which make insufficient the efficiency of gene transfer for the desired application (Dizaj et al., 2014). However, modifications of the nanoparticle surface, such as PEGylation or addition of zwitterionic molecules, have been used to delay the clearance (Shi et al., 2021). The third class of nanomaterials is represented by inorganic particles, which includes: gold-nanoparticles, carbon-dots (CDs), silica nanoparticles, iron-oxide magnetic nanoparticles, and spherical nuclei acid nanoparticles (SNA, NPs). While they are less expensive and easier to produce, the big challenges of these nanomaterials are their biodistribution and neurotoxicity (Guo et al., 2021). As for viral delivery, nanoparticles can be directed into the CNS systematically or locally, therefore sharing the same advantages and concerns already discussed for both routes. Of note, systemic delivery of nanoparticles was proven successful in trespassing the BBB when modifications such as PEGylation and conjugation to transferrin were applied (Huang et al., 2007; Gregory et al., 2020). Local delivery, which include intrathecal (IT), intracerebroventricular (ICV), and intranasal injection although more invasive, offers the advantage of bypassing the BBB. The ICV route delivers the particles through the cerebral ventricles with the help of a device consisting of a dome and a catheter that are implanted under the scalp (Duma et al., 2019). The IT injection, instead, takes place into the CSF of the lower spinal cord (Fowler et al., 2020). Finally, in intranasal injection the particles are delivered into the olfactory bulb that has direct access to the CNS (Bryche et al., 2020). Although this route is the less invasive compared to the previous ones, it has also the lowest delivery rate. Clinical trials based on gene delivery of non-viral particles-based (i.e., antisense oligonucleotide or RNA interference) have been employed for the treatment of neurodegenerative disorders such as spinal muscular atrophy (NCT04591678), Parkinson’s disease (NCT03976349) and Alzheimer’s disease (NCT03186989). However, most of the studies exploring nanoparticle were focused on the treatment of brain cancer, especially glioblastoma, given the lethality of the grade IV of this tumor. Recently, a phase 0 clinical trial (NCT03020017), that utilized gold nanoparticles packed with siRNAs targeting the *Bcl2L12* oncogene (Kumthekar et al., 2021), showed interesting results in patients after systemic delivery. As the field is exponentially growing, advanced approaches aim at ameliorating nanoparticle stability, batch-to-batch consistency, and neurotoxicity: the objective is to render their use feasible for clinical trials of neurological disorders. We believe that nanoparticles delivery holds tremendous promises that could be pursued also for therapeutic treatments of RTT.

## Discussion

The ground-breaking idea that RTT is not an irreversible condition boosted studies investigating the beneficial effects of advanced gene and RNA therapies. In the last decade, reversion of disease symptoms upon *MECP2* re-expression was exponentially reported by several research groups. In spite of positive observations, the dosage-sensitivity of *MECP2* gene complicated the feasibility of this approach: reduced levels of MeCP2 lead to RTT-like phenotypes, likewise its overexpression results in neurological defects. The situation could be even more hard to manage in female patients who already express normal levels of WT MeCP2 in approximately 50% of transduced cells; perhaps the expression of the transgene in these cells will result in gain of function phenotypes (Takagi, 2001). Moreover, RTT patients generally carry hypomorphic mutations which determine a partial loss-of-function of MeCP2, therefore increasing the risk for complications due to protein overexpression. Accordingly, whereas most of the gene therapy studies have been conducted in a *Mecp2* null genetic background, two recent studies evaluated *MECP2* gene therapy benefits in mouse models harboring either the truncated mutation R294X (Collins et al., 2022) or the missense mutation R133C (Vermudez et al., 2022). When a wild-type copy of *MECP2* was supplied to male animals, most of RTT features were rescued and, importantly, no “over-expression phenotype” was observed. In contrast, similar experiments performed in heterozygous female mice resulted in behavioral tasks associated with adverse effects of MeCP2 supplementation (Pitcher et al., 2015; Collins et al., 2022), confirming the risk of transducing high levels of *MECP2* in this gender. This study also suggested the importance of evaluating partial loss-of-function alleles in female mouse models, in addition to the vastly used null models, and including in pre-clinical studies the assessment of behavioral abnormalities associated with MeCP2 overexpression.

After years of refinement protocols for *MECP2* gene therapy, remarkable news came from the study named NGN-401 by Stuart Cobb, the chief scientific officer of Neurogene.^1^ Cobb’s team used the Expression Attenuation via Construct Tuning (EXACT) technology to deliver AAV9:*MECP2* directly into the cerebrospinal fluid of male mice. EXACT technology enables to self-regulate the transgene expression by tuning *MECP2* levels within a “safe range,” thus avoiding the toxicity associated with its overexpression. Male mice treated with a high dose of transducing particles (3 × 10^11^) extended their lifespan to approximately 37 weeks, while heterozygous females did not show any sign of toxicity. Encouraging results of NGN-401 were obtained also in non-human primates. Thus, very recently (24th January, 2023), Neurogene announced the clearance by FDA to initiate a clinical trial of the investigational gene therapy, NGN-401, in female children with RTT.^2^ In similar way, Steven Gray and Sarah Sinnet at UT Southwestern Medical Center used the mi-RARE platform to safely control the level of MeCP2 protein upon its delivery. After promising results in preclinical studies (as described above), a gene therapy program, named TSHA-102, has initiated the first clinical trial – the REVEAL Adult study – in females of 18 years or older living with RTT. This study will evaluate the safety, tolerability and preliminary efficacy of a single intrathecal administration of TSHA-102 into the spinal fluid^3^ of affected girls.

**Figure 5:**
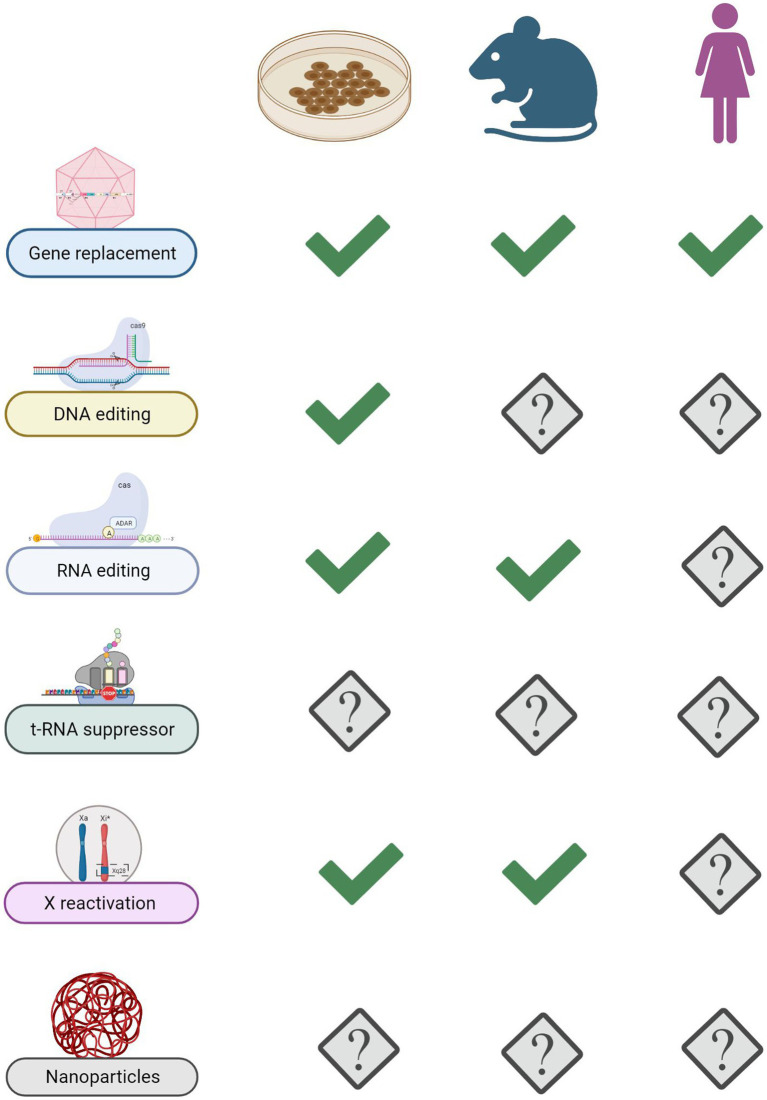
State of the art of advanced RTT therapies. Summary of progresses made with various genetic approaches in *in vitro* and *in vivo* models of disease. Gene replacement therapy stands out as the only one so far that reached RTT girls.

An important advance in the field will probably derive from genome editing that, despite some concerns, appears the most promising and definitive approach to restore physiological levels of functional MeCP2. Direct correction of the endogenous mutant *MECP2* would in fact bypass risks of toxicity and inflammation related to MeCP2 overexpression. However, both approaches, conventional gene therapy and genome editing, are limited by the low tropism of most viruses. In this regard, more studies should be addressed to reveal which brain area have a more detrimental role from unbalanced levels of MeCP2 and therefore need an urgent intervention. The objective might be a localized therapeutic intervention to recover more severe disease symptoms. Beside the genetic approaches so far explored and summarized in Figure 5, several research groups attempted to normalize the downstream pathways altered in RTT. Even though many approaches were proved successful in preclinical studies, sadly, only one completed a phase 3 clinical trial.^4^ Indeed, oral administration of Trofinetide, analog of the amino-terminal tripeptide of insulin growth factor 1 (IGF-1), in children and adolescent with RTT, improved primary and secondary clinical endpoints.^5^ The difficulty in finding effective therapies, in spite of numerous promising pre-clinical trials might imply that we must adhere more strictly to optimal rules (Katz et al., 2012) and use ideal cellular models for drug screening such as patients’ iPSC-derived neurons and organoids. Indeed, neurons derived from human MeCP2 deficient iPSCs recapitulated deficits previously observed in mouse primary neurons and human RTT brain (Marchetto et al., 2010; Farra et al., 2012; Haase et al., 2021). Since these cells offer an unlimited source and various genetic backgrounds, iPSCs hold enormous promise for drug discovery. Considering animal models, we believe in the necessity to establish whether models recapitulating human pathogenic mutations, or the null line are equally suitable for preclinical trials, which gender should be treated, and in general, which is the best time frame to assess efficacy.

## Author contributions

NL conceived the idea of this review. NL and MP wrote and refined together the manuscript. DP produced all the schematic illustrations for the figures. All authors contributed to the article and approved the submitted version.

## Funding

This work is supported by the Italian parents’ association “Pro RETT Ricerca” to NL and by the “European Union’s Horizon 2020 research and innovation programme under the Marie Skłodowska-Curie grant agreement No.845992” to MP.

## Conflict of interest

The authors declare that the research was conducted in the absence of any commercial or financial relationships that could be construed as a potential conflict of interest.

## Publisher’s note

All claims expressed in this article are solely those of the authors and do not necessarily represent those of their affiliated organizations, or those of the publisher, the editors and the reviewers. Any product that may be evaluated in this article, or claim that may be made by its manufacturer, is not guaranteed or endorsed by the publisher.

## Footnotes

^1^https://rettsyndromenews.com/news/non-toxic-gene-therapy-rett-syndrome-extends-lifespan-mice/

^2^https://rettsyndromenews.com/news/rett-gene-therapy-ngn-401-cleared-advance-clinical-trials/?cn-reloaded=1link

^3^https://clinicaltrials.gov/ct2/show/NCT05606614?cond=rett+syndrome&draw=2&rank=3

^4^https://clinicaltrials.gov/ct2/show/NCT04181723

^5^https://acadia.com/media/news-releases/acadia-pharmaceuticals-announces-positive-top-line-results-from-the-pivotal-phase-3-lavender-trial-of-trofinetide-in-rett-syndrome/
